# A Rare Case Report of Acute Necrotizing Encephalopathy of Childhood

**Published:** 2020

**Authors:** Seyed Ahmad HOSSEINI, Fatemeh Sadat TABATABAEI, Mohammad Hadi MOLSEGHI, Hamed JAFARPOUR, Amir Hossein GOUDARZIAN, Arash REZAEI SHAHMIRZADI

**Affiliations:** 1 Children’s and Neonatal Health Research Center, Golestan University of Medical Sciences, Gorgan, Iran.; 2 Clinical Research Development Unit (CRDU), Student Research Committee, Golestan University of Medical Sciences, Gorgan, Iran; 3 Medical Student, Student Research Committee, Mazandaran University of Medical Sciences, Sari, Iran; 4 Student of Psychiatric Nursing, Student Research Committee, Mazandaran University of Medical Science, Sari, Iran

**Keywords:** ANEC, Encephalopathy, Thalamus, Brain stem, Children

## Abstract

**Objective:**

Acute necrotizing encephalopathy of childhood (ANEC) is a fast growing disease, accompanied by progressive encephalopathy. The aim of this study was to report a rare case of ANEC in a four-year-old boy with bilateral thalamic necrosis and non-fatal outcomes.

**Case Report:**

The patient was a four-year-old Iranian boy, without any history of health problems or hospitalization, except for jaundice and phototherapy in the neonatal period. He had no neurological signs or symptoms during admission, and he was admitted only with chief complaints of acute onset of fever, coryza, and icterus. In the neurological consultation, brain MRI was requested to analyze the possibility of brain damage. The results indicated the involvement of cerebellum, thalamus, and basal ganglia, which led to the diagnosis of ANEC.

**Conclusion:**

Based on our findings, although ANEC is a rare disease, it should not be underestimated.

## Introduction

Acute necrotizing encephalopathy of childhood (ANEC) is a fast growing disease, accompanied by progressive encephalopathy and seizures (1). ANEC is a rare disease, which mostly occurs in children with no history of health problems. It is associated with fever, seizure, mental disorder, and high risk of mortality. Clinical findings of ANEC are mostly non-specific and include increased cerebrospinal fluid (CSF) protein content, increased liver enzyme levels, and thrombocytopenia (2, 3).

Radiological signs of ANEC, which are exclusive and diagnostic, include multifocal and symmetrical involvement of the thalamus, brainstem, supratentorial region, white matter, and cerebellum (4). Affected children usually exhibit severe neurological deficits and a wide range of long-term mental problems (3). Also, impaired liver function is common among these patients (5). Effective factors in this disease include viral infections (1).

Differential diagnoses of ANEC include Reye syndrome, leishmaniasis (hyperammonemia, hypoglycemia, and metabolic acidosis), acute disseminated encephalomyelitis (CSF pleocytosis as a more common unilateral mental disorder), and Japanese encephalitis (common involvement of the cortex and dark matter) (6). ANEC was first reported in Japan in 1995 (2). This disease has been rarely reported in Eastern Asia. Nevertheless, 240 cases were reported in Asia, five in north America, and ten in Europe (7). Generally, treatment of these patients includes strengthening of the immune system and hypothermia (8). 

In this case report, we describe the clinical and neuropathologic findings of a four-year-old boy with bilateral thalamic necrosis and non-fatal outcomes.

## Case report

The patient was a four-year-old Iranian boy, with no history of health problems or hospitalization, except for jaundice and phototherapy in the neonatal period. He had been immunized before the emergence of signs, according to the Iranian immunization program and guidelines (2015), and showed normal growth. The parents were not consanguine, and family history of neurological disorders was negative. He was admitted to the hospital with acute onset of fever and symptoms of coryza and otitis media in 2016.

Immediately after admission, the patient’s vital signs were measured: blood pressure, 110/80 mmHg; pulse rate, 130 bpm; respiratory rate, 32 bpm; and temperature, 39.3°C. Examinations revealed that the patient was conscious, aware of time and place, and able to identify his family and obey orders. The cranial nerve examination was normal, and deep tendon reflexes (DTRs) were in the normal range (1+ to 2+). On the other hand, he presented with scleral icterus. The tympanic membrane was inflamed and bulged, and cardiac and pulmonary auscultations were abnormal. Examination of other organs showed normal results. 

The patient had no history of trauma, food poisoning, possible drug toxicity, or hospitalization. Symptoms of loss of consciousness emerged four to six hours after hospitalization, followed by an increase in body temperature, rhinitis, and cough. He was irritable and unable to stay awake, speak properly, or obey instructions. He also showed no proper response to environmental stimuli. In addition, blurred vision, decreased muscle tone and force, increased DTRs (3+ to 4+), and bilateral Babinski sign were reported. On the following day, multiple generalized tonic-colonic seizures occurred. 

The results of serological, parasitological, and urinary analyses were normal. Also, liver function was evaluated in the patient. Based on the results, a high level of liver enzymes was reported: alanine transaminase (ALT), 42 IU/L; aspartate transaminase (AST), 56 IU/L; and alkaline phosphatase (ALP), 210 IU/L. In the cardiac assessment, echo and electrocardiography (ECG) findings were normal, and possibility of cardiac problems, especially endocarditis, was ruled out. The CSF examination indicated the following results: white blood cell count (WBC), 0-3; red blood cell count (RBC), 1-2; glucose level, 65 mg/dL; and protein level, 45 mg/dL. Moreover, all cultures were negative; consequently, encephalitis and infectious disease were ruled out. Also, hepatic encephalopathy was ruled out based on the gastrointestinal consultation. 

**Figure 1:**
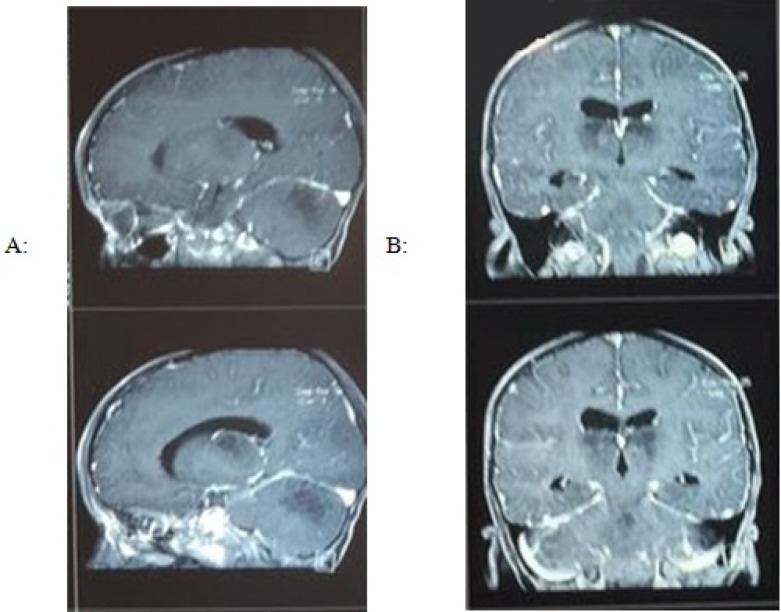
Sagittal (A) and coronal (B) views of brain MRI of thalamic lesion

In the neurological consultation, brain MRI was requested for the patient to analyze the possibility of brain damage. The results indicated the involvement of cerebellum, thalamus, and basal ganglia, which led to the diagnosis of ANEC (Figure 1). Methylprednisolone (20 mg/kg/day for seven days) was prescribed by the neurologist. On the third day, the patient’s fever was controlled, whereas his consciousness level was low, and he was unable to open his eyes. After seven days of treatment, he opened his eyes, and the pupils were responsive to light. Since he had oral lesions (oral thrush), nystatin was prescribed at a dose of 20 drops every six hours, methylprednisolone was discontinued, and oral prednisolone (5 mg/kg/day) was administered instead of corticosteroid pulse therapy.

About 12 days after admission, the patient’s level of consciousness improved, while his eyesight remained poor. Accordingly, optical examination was carried out, which indicated the possibility of cortical blindness. About 20 days after admission, his general condition improved, and he was able to speak; his level of consciousness was also satisfactory. Following that, he was transferred to the ward. Since he was diagnosed with strabismus in the follow-up, involvement of the sixth nerve was considered.

## Discussion

Diagnosis of ANEC in children is usually established based on an acute neurological disorder. The symptoms are mostly non-specific, while paraclinical signs and MRI findings are used to confirm the diagnosis (2). Children with ANEC usually have no history of serious health problems, similar to our case (7). Generally, ANEC is divided into two major categories. The first type is caused by pathogens of previous infections, such as encephalopathy induced by influenza, herpesvirus (HHV-6), and rotavirus. 

Acute necrotizing encephalopathy type 1 is inherited with an autosomal dominant pattern (9). Missense mutations in the gene encoding the nuclear pore protein (RAN binding protein 2) were recognized as susceptibility alleles for familial and recurrent ANEC; this type of acute necrotizing encephalopathy was called “ANE1” (10). Individuals with ANE1 develop lesions in certain regions of the brain. As the condition progresses, these brain regions develop swelling (edema) and bleeding (hemorrhage), followed by tissue death (necrosis). It is estimated that half of individuals with ANE1 are susceptible to recurrent episodes and may develop a subsequent infection, resulting in neurological decline. Some people may experience numerous episodes throughout their lives. Neurological function deteriorates following each episode as more brain tissue is damaged. Progressive brain damage and tissue loss are known to cause encephalopathy (11).

The second type of ANEC is diagnosed based on clinical and medical imaging findings (10). In both types of ANEC, MRI findings are useful. The center of thalamic lesions usually shows perivascular hemorrhage, as well as necrosis of neurons and glial cells, corresponding to slightly high signals on apparent diffusion coefficient (ADC) maps. The peripheral regions show congestion of arteries, veins, and capillaries, in addition to acute swelling of oligodendrocytes, corresponding to low signals in the surrounding areas, with extravasations on the edge of thalamic lesions due to high signals in the outermost region (12). Our patient had no history of serious infections and was admitted to hospital with symptoms of coryza and otitis media. All serological and parasitological results were normal, and diagnosis was established based on the clinical examination and medical imaging.

The mortality rate of ANEC has been estimated at nearly 14%. Moreover, 83% of patients with ANEC develop neurological disorders (8). Involvement of the cerebellum, thalamus, and basal ganglia is suggestive of neurological damage, as reported in the MRI of our patient. Although ANEC is associated with mortality and severe neurological damage in most patients (13), timely diagnosis and treatment led to the successful treatment of our patient. The only neurological damage, despite serious efforts to control and treat the disease, was damage to the sixth nerve, which resulted in strabismus.

**In Conclusion, **Craniofacial syndromes, because of their wide spectrum of signs and symptoms, pose a diagnostic challenge for clinicians. Therefore, a systematic approach is necessary for accurate diagnosis of these syndromes with different manifestations. The signs and symptoms of our patient fulfilled all of the diagnostic criteria for ANEC. Considering the cost of paraclinical tests, they can be postponed for patients with low economic resources.

The authors would like to thank the patient for his cooperation.
